# Appropriate Timing of End-of-Life Care: A Dutch Policy Analysis and Opportunities for Improvement

**DOI:** 10.1089/pmr.2023.0087

**Published:** 2024-07-19

**Authors:** Wim J. J. Jansen, Jos G. C. Lerou, Patrick R. Schober, Karolina M. Szadek, Bregje A. A. Huisman, Monique A. H. Steegers

**Affiliations:** ^1^ Dept. Anesthesiology, Amsterdam UMC, Amsterdam, Netherlands.; ^2^ Hospice Kuria, Amsterdam, the Netherlands.

**Keywords:** costs of care, end-of-life, health care costs, health insurance reimbursement, life expectancy, survival, terminal care

## Abstract

**Background:**

The Exceptional Medical Expenses Act (EMEA) guaranteed public financing for the costs of end-of-life care in The Netherlands until 2015. A life expectancy shorter than three months was a prerequisite for a patient to qualify.

**Objective:**

To estimate survival and its potential predictors using the start date of EMEA funded end-of-life care as time origin, and to calculate the ensuing costs.

**Design:**

Retrospective observational study using data retrieved from multiple datasets of the national statistical office Statistics Netherlands (https://www.cbs.nl/en-gb/).

**Setting:**

Included were all adult patients, who received EMEA funded end-of-life care in hospice units in nursing homes and homes for the elderly in The Netherlands between January 1, 2009, and December 31, 2014.

**Results:**

In 40,659 patients (median age 79 years), the distribution of survival was extremely skewed. Median, 95%, and maximum survival times were 15 (95% confidence interval [CI] = 15–15), 219 (210–226), and 2,006 days, respectively. The 90-day and 180-day survival rates were 12.4 (12.1–12.7)% and 6.2 (6.0–6.5)%, respectively. Although age, gender, diagnosis, and start year of end-of-life care were statistically significant independent predictors, clinical significance is limited. End-of-life care was delivered for a total of 1,720,002 days, costing almost 440 million Euros. Fifty-nine percent of the costs was for barely 11% of patients, i.e., those who received end-of-life care for more than 90 days.

**Conclusion:**

The use of life expectancy is a weak basis for the appropriate timing of end-of-life care. Further research should evaluate potential tools to improve the timing of end-of-life care, while using available resources efficiently.

## Introduction

There is no standard criterion to define the terminal phase of life.^1^ Notwithstanding, the physician’s estimation of life expectancy may be required if the patient is to qualify for appropriate end-of-life care.**^2^** In the United States, the Medicare program requires physicians to make a prognosis of a six-month life expectancy for a patient to be eligible for hospice care.^3^ Hospice enrollment can be continued if the patient’s prognosis is again estimated to be a six-month life expectancy. The Dutch Health Care System organizes medical care as a “continuum of care”: Curative care diminishes, whereas palliative care increases from conception to death. Good timing is thereby a prerequisite for appropriate care. At a certain moment, patients may suffer from a life-limiting disease and may need a kind of end-of-life care they cannot afford without extra reimbursement of the costs.

The Exceptional Medical Expenses Act (EMEA) guaranteed public financing for the costs of end-of-life care in The Netherlands until 2015, allowing everyone to claim end-of-life care, not only in a hospice but also at home.^4^ In contrast with Medicare eligibility criteria, the EMEA did not require that patients gave up certain curative interventions to qualify for end-of-life care.

By providing funding for palliative care services, the EMEA helped people with life-limiting illnesses receive the support they needed to live as comfortably and fully as possible. Under the EMEA, the type, and hours of care were laid down in so-called “Care Packages.” The rates for each of the care packages were determined annually.^5–10^ Care in the terminal phase of life was the most comprehensive care package with a total of 30 hours of care per week, including nursing care (11 hours), medical care (two hours), personal care (13 hours), supportive assistance (three hours), and day care (one hour). However, the condition to receive such a package was that life expectancy was shorter than three months. Once given, the eligibility was valid until death.

In contrast with the putative life expectancy of three months, we noticed in clinical practice that patients admitted for end-of-life care to in-patient hospices of the Amsterdam region died within three weeks on average. This raised the question whether the start of the EMEA end-of-life care package had been well-timed. Accordingly, our main goal was to determine the survival time in a large sample of the Dutch population using the start date of EMEA funded end-of-life care as time origin. Therewith we investigated if the following variables were clinically significant predictors for survival time: age, gender, diagnosis, and the calendar year patients qualified for end-of-life care. Our secondary goal was to assess the costs related to this EMEA care package, especially the distribution of costs among patients.

## Methods

We performed a retrospective study using data collected and provided by Statistics Netherlands, i.e., “*Centraal Bureau voor Statistiek”* (CBS) (https://www.cbs.nl/en-gb). A personal identification number uniquely identifies each person in each of the CBS databases. Further details about CBS are in the data sharing statement.

### Research ethics

The study did not fall under the scope of the Medical Research Involving Human Subjects Act (WMO) as the subjects were not physically involved in this retrospective file research. Consequently, and according to the regulations of the VU University Medical Center, approval by its Medical Ethics Review Committee was not required. Data protection, confidentiality, and anonymity are guaranteed by adopting the rules of CBS.

### Data handling

All databases provided by CBS had the format of the Statistical Package for Social Sciences (IBM^®^ SPSS^®^). The personal identification numbers allowed to treat the data as relational data. Hence, we first imported the databases of interest into data frames in **R** (version 4.1.0; The **R** Foundation for Statistical Computing, Vienna, Austria).^11^ Then, we merged the relational data in a single database using the personal identification number as key. It contained all persons who used the EMEA end-of-life care package in the last six years of the existence of the EMEA, i.e., between January 1, 2009, and December 31, 2014.

The final database contained primary and calculated variables. Primary variables were as follows: gender, date of birth, date of death, the dates when end-of-life care started and stopped, calendar year in which the eligibility started, cause of death according to the 10th iteration of the International Classification of Diseases (ICD-10 code), and main basis for eligibility, e.g., somatic or psychogeriatric. Calculated variables were as follows: age, survival time, and number of days of end-of-life care. Time origin to determine survival time was the date of the first day of eligibility for end-of-life care. Survival time was not always equal to the number of days with end-of-life care (see Costs).

In addition, there was one grouping variable. Causes of death were first grouped as either “malignancy” (ICD-10 codes: C00-D48) or “nonmalignancy” (all other ICD-10 codes). The latter two variables were then combined with the “main basis for eligibility” in such a way that one grouping variable with three categories remained: somatic/malignancy, somatic/nonmalignancy, and psychogeriatric.

### Data verification

In total 40,659 persons were included in the study. Although we had obtained 41,261 unique “cases,” 602 (1.5%) of them were excluded because of age <18 years, “missing values” for one or more variables, a main basis for eligibility other than somatic or psychogeriatric or a changing basis for eligibility, or left-censoring, i.e., the person qualified for end-of-life care before January 1, 2009.

### Costs

We calculated the costs of care per day per patient using the annually changing rates for the end-of-life care package.^5–10^

We differentiated costs between patients who received end-of-life care for either less or more than 90 days. This criterion reflects the fact that a life expectancy of less than three months is required to be eligible for end-of-life care in The Netherlands.

To calculate costs, we used the actual number of days patients effectively received end-of-life care, not the days of survival time. It turned out that the duration of eligibility (= number of days of reimbursed end-of-life care) was not equal to the survival time in 2384 persons (6%). Three causes for this difference were as follows: 1) there was one period of end-of-life care, but eligibility ended before the date of death, 2) there were several periods of end-of-life care and the end date of the last period coincided with the date of death, and 3) there were several periods of end-of-life care and the last period ended before the date of death.

### Statistical analysis

Descriptive summary statistics are presented as median (IQR) [minimum–maximum], unless stated otherwise.

We chose to estimate the association between survival time and four potential predictor variables: age, gender, start year of eligibility, and the combination of basis for eligibility and diagnosis. For the latter combination, we had to assume that the ICD-10 codes grouped as either ‘malignancy’ or “nonmalignancy” reflected the diagnosis at the time origin. Age was the age at time origin, not at the date of death.

Survival rates were estimated using the Kaplan–Meier method. In addition, we assessed length of survival in relation to only one of the potential predictors at a time, ignoring the simultaneous effects of the other three.

A multivariable Cox proportional hazard (PH) regression model was used to study the independent relationship between each of the four potential predictor variables and survival time, while simultaneously adjusting for potential confounding effects by the other predictor variables.^12,13^ Age was introduced as continuous covariate in the Cox PH model.

We performed all analyses in **R**, using *inter alia* the survival package. A *p* value < 0.05 was considered statistically significant.

### Sensitivity analysis

We studied the relative importance of the 2384 (6% of total) persons in whom the number of days with end-of-life care was smaller than the survival time. Therefore, we excluded them from analysis and compared the results with those obtained with all persons.

## Results

Table 1 describes the main characteristics of the total of 40,659 patients. Data on 848 (2.1%) patients who were still alive on December 31, 2014, were included as right-censored data in the survival analysis.

**Table 1.** Characteristics of the Patients in the Study Sample (*n* = 40,659)

	Median (IQR) [min-max] or *N* (%)
Age (years)	79 (70–86) [19–106]
Gender	
Male	19,464 (48%)
Female	21,195 (52%)
Start year eligibility	
2009	6,363 (15.6%)
2010	6,693 (16.5%)
2011	6,735 (16.6%)
2012	6,915 (17.0%)
2013	6,947 (17.1%)
2014	7,006 (17.2%)
Main basis for eligibility and diagnosis (ICD-10)	
Somatic and malignancy	26,907 (67.6%)
Somatic and non-malignancy	12,263 (30.2%)
Psychogeriatric	641 (1.6%)
Unavailable ICD-10	848 (2.1%)^a^

^a^ Data from these patients were treated as right-censored data because they were alive on December 31, 2014.

### Survival

**FIG. 1.**
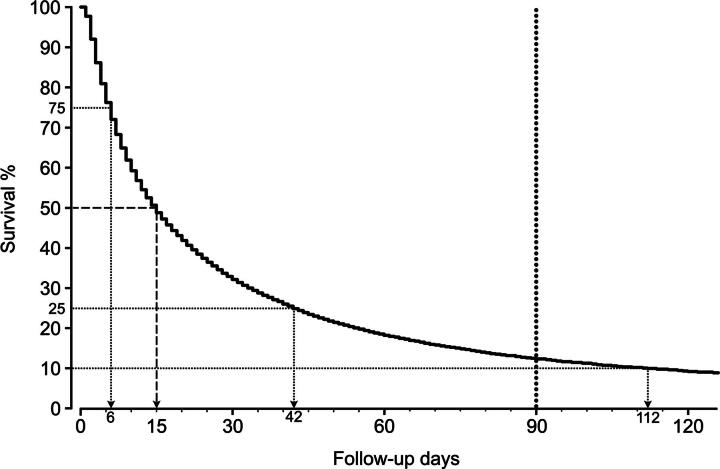
Kaplan–Meier survival curve showing survival probability following start of end-of-life care (*n* = 40,659). The distribution of survival time among patients was extremely positively skewed with a median and maximum survival time of 15 and 2,006 days, respectively. The curve is therefore truncated at 120+ days to show more details for survival probabilities between 10% and 100%. The vertical dotted line at 90 days is the boundary line between a survival time shorter or longer than the maximum life expectancy estimated by a physician. The survival rate is only 12.4% at 90 days.

The distribution of survival time among patients was extremely positively skewed. Median survival time was 15 (95% confidence interval [CI] = 15–15) days; 75%, 90%, 95%, or 100% of the patients deceased within 42 (42–43), 112 (109–116), 219 (210–226), or 2,006 (no 95% CI) days, respectively. Figure 1 shows the Kaplan–Meier survival curve truncated at 120+ days. Table 2 shows the survival rates for six specific survival times in the range 10–180 days. The 90-day and 180-day survival rates were 12.4 (95% CI = 12.1–12.7)% and 6.2 (6.0–6.5)%, respectively.

**Table 2.** Survival Rates for Various Survival Times (*n* = 40,659)

Survival time (days)	Survival rate (95% CI) (%)
10	59.2 (58.7–59.7)
15	48.8 (48.3–49.3)
30	32.1 (31.6–32.6)
60	18.2 (17.8–18.6)
90	12.4 (12.1–12.7)
180	6.2 (6.0–6.5)

**FIG. 2.**
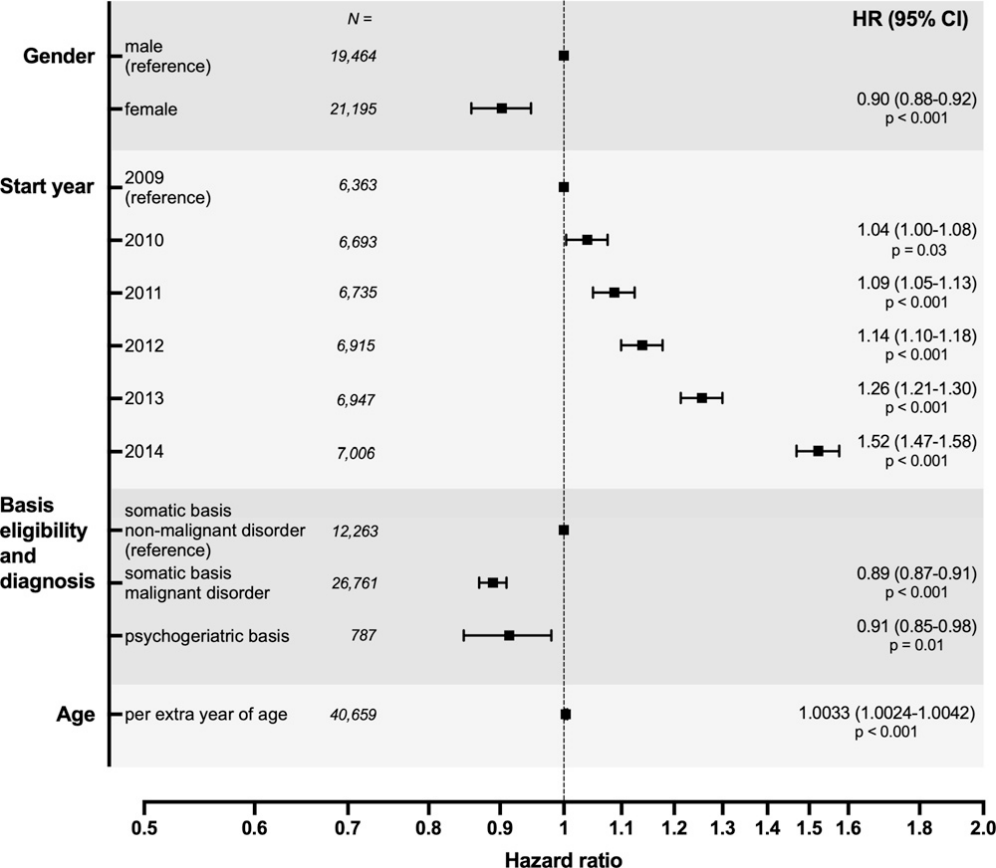
Forest plot of results of Cox proportional hazards regression analysis that tests the independent relationship between each of the four potential predictor variables (gender, start year of end-of-life care, basis for eligibility and diagnosis, and age) and survival time, while simultaneously adjusting for potential confounding effects by the other predictor variables. Age was introduced as continuous covariate. HR = hazard ratio.

Table 3 shows median survival times in our study sample calculated separately with respect to the potential predictors. Figure 2 shows that each of the potential predictors was independently and significantly associated with survival after adjusting for the other predictors. Females had a statistically significant lower hazard rate of dying than males (HR = 0.90 [95% CI = 0.88–0.92]). The HR = 0.90 for gender implies that the hazard rate of dying at any given time is 10% lower for females than for males. With 2009 as reference, HR increases from 1.04 (95% CI = 1.00–1.08) in 2010 to 1.52 (1.47–1.58) in 2014. Patients with a malignancy had a statistically significant lower hazard rate of dying than those suffering from a nonmalignant disease (HR = 0.89 [95% CI = 0.87–0.91]). As HR for age was 1.0033 per extra year of age, the hazard of death increased with 3.3% per decade of age (age was determined at the time origin).

**Table 3.** Median Survival Times in the Study Sample Calculated Separately in Relation to Four Potential Predictor Variables (*n* = 40,659)

Potential predictor variable	Median (95% CI) (days)
Gender	
Male	14 (13–14)
Female	16 (16-16)
Age	
<= 79 years	17 (16–17)
>79 years	13 (13-13)
Start year	
2009	18 (17–19)
2010	17 (16–17)
2011	15 (15–16)
2012	14 (13–15)
2013	13 (12–14)
2014	13 (12–13)
Basis for eligibility and diagnosis ^a^	
Somatic and nonmalignancy	9 (9–9)
Somatic and malignancy	17 (17–18)
Psychogeriatric	9 (8–10)

^a^ 848 observations out of 40,659 deleted due to missing data (right-censored data).

### Costs

End-of-life care was delivered for a total of 1,720,002 days in the six-year period of the study. The distribution of survival time among patients was extremely positively skewed. Accordingly, the average number of days with end-of-life care was 42.3 (SD = 93.6) days, but the median number was 14 (5–39) [1 to 1701] days.

Based on the annual rates of the EMEA, nearly 440 million Euros were reimbursed in the period 2009–2014 (Table 4). Only 41% of the total costs has been spent on 89% of the patients, i.e., those who received end-of-life care for 90 days or less. As a corollary, 59% of the costs was for barely 11% of patients, i.e., those who received end-of-life care for more than 90 days. Part of the latter persons was contributing to costs year after year, as shown by the differentiated figures in Table 4.

**Table 4.** Costs for End-of-Life Care in The Netherlands During the Years 2009 to 2014 Under the Exceptional Medical Expenses Act

			Costs^a^ per calendar year (k€)^b^						
Start year of eligibility^c^	Duration of eligibility^d^ (days)	*N* ^e^	2009	2010	2011	2012	2013	2014	Total (% grand total)
2009	≤90	*5,546*	25,825	1,851	5	0	0	0	27,681 (6.3%)
	>90	*817*	26,210	14,712	2,283	600	254	0	44,058 (10.0%)
2010	≤90	*5,871*	0	26,694	1,567	0	0	0	28,261 (6.4%)
	>90	*822*	0	27,671	19,639	4,724	1,587	1,116	54,737 (12.4%)
2011	≤90	*5,958*	0	0	27,319	1,596	0	0	28,915 (6.6%)
	>90	*777*	0	0	25,150	18,555	4,491	1,911	50,107 (11.4%)
2012	≤90	*6,163*	0	0	0	29,650	1,497	8	31,156 (7.1%)
	>90	*752*	0	0	0	27,602	16,003	3,723	47,328 (10.8%)
2013	≤90	*6,265*	0	0	0	0	29,250	1,825	31,075 (7.1%)
	>90	*682*	0	0	0	0	26,014	15,341	41,355 (9.4%)
2014	≤90	*6,490*	0	0	0	0	0	31,888	31,888 (7.3%)
	>90	*516*	0	0	0	0	0	23,388	23,388 (5.3%)
	Totals	*40,659*	52,035	70,928	75,963	82,727	79,097	79,201	439,950 (100%)

^a^ Costs were calculated using the annually changing tariff, i.e., 234.51 € (2009), 238.65 € (2010), 243.20 € (2011), 266.05 € (2012), 270.24 € (2013), and 277.97 € (2014) per day of end-of-life care.

^b^ 1 k€ = 1 kilo Euro = 1000 €.

^c^ Costs calculations were differentiated for the years in which patients became eligible for end-of-life care.

^d^ Costs were differentiated between patients who received end-of-life care for a period either shorter or longer than the “90-day criterion,” reflecting the fact that a life expectancy of less than three months is required to become eligible for end-of-life care in The Netherlands.

^e^ Number of patients are in italics.

### Sensitivity analysis

We obtained similar results with or without the 2384 persons in whom the actual number of days with end-of-life care was smaller than the survival time.

## Discussion

### Main findings

We studied the survival after the onset of end-of-life care and the ensuing costs in all persons who were insured under the Exceptional Medical Expenses Act and eligible for the care package “care in the terminal phase of life” in The Netherlands between January 1, 2009, and December 31, 2014. Patients qualified for end-of-life care because a physician estimated that their life expectancy was shorter than 90 days. Our nationwide study shows that the distribution of survival was extremely skewed to the right with a median survival time of only 15 days (Fig. 1), indicating that a significant number of patients received end-of-life care during a very short period. Age, gender, start year of end-of-life care, and diagnosis were all independent, statistically significant predictors for survival time. However, the absolute effect sizes were rather small and clinical significance poor. The number of patients deemed eligible for end-of-life care grew annually from 6,363 in 2009 to 7,006 in 2014. In total, almost 440 million Euros was reimbursed over the six-year period, but the majority of the money was needed for a small minority of patients.

Although the survival time of 87.6% of all patients was in accordance with the Dutch requirement of a three-month life expectancy to qualify for end-of-life care (Table 2), half of the patients died within only 15 days. The latter finding matches figures from the United States, showing a median survival of 18 days^14^ and a median length of stay in a hospice of 16–20 days.^15–16^ These very short periods of end-of-life care reflect the poor prognostic accuracy of physicians. In patients with advanced cancer, a review showed that clinicians overestimated patients’ survival in 12 studies, whereas only five studies showed underestimations.^17^ In noncancer patients, general practitioners recognized approaching death only at a late stage in the disease trajectory.^18^ When the moment of death was nearby, the accuracy of predicting life expectancy increased.^19–21^

We may point out potential determinants of the physicians’ prognostic accuracy. A strong doctor–patient relationship is associated with overoptimistic predictions of survival time.^19^ Consequently, patients may postpone to recognize that end of life is nearby, thus delaying what might be the appropriate care.^22^ Moreover, patients and professionals alike may avoid open discussions until disease is advanced and patients are only left a late referral to end-of-life care.^22^ Furthermore, cancer patients have a typical steep functional decline just before death. This may help to explain the clinicians’ overestimations of life expectancy. Median survival was indeed only 26 days in 313 cancer patients referred to an outpatient hospice program.^23^

Our results regarding the predictors’ gender, age, and diagnosis are in line with those of Harris and co-authors.^14^ However, they found a six-month survival rate of 13.4%, whereas we found that 90-day and 180-day survival rates were 12.4% and 6.2%, respectively.

The increasing number of patients we found for the period 2009–2014 reflects the growing attention for and the structural funding of palliative care in The Netherlands since 1997. The capacity for end-of-life care in hospices and hospice units in nursing homes and homes for the elderly has also increased.^24^

Only 11% of the patients consumed 59% of the total number of days and costs. The possibility to be eligible for more than 90 days is a direct consequence of the government’s decision that a given eligibility for end-of-life care is valid until death.

### Strength and limitations of the study

A strength is that we included all patients to whom end-of-life care was delivered in The Netherlands under the EMEA in six consecutive years. Therefore, the study does not suffer from possible selection bias related to nonrandom samples of patients.

A limitation is that we could not include any data after 2014 because the care provided under the EMEA was terminated at the end of 2014. This care is now covered by the Long-term Care Act and the Dutch Health Insurance Act.^25^ Recently the criterion of a life expectancy of less than three months was skipped by the Dutch Ministry of Health, Welfare and Sport.^26^ However, it is unclear how health care insurance companies will deal with this decision in reimbursing end-of-life care.

Strictly speaking, we did not know the patient’s diagnosis, which we used as a predictor, at the onset of end-of-life care. Therefore, we used the ICD-10 causes of death recorded by CBS as a surrogate diagnosis at the time of origin. We are convinced that categorizing the ICD-10 code in only two broad categories, i.e., malignancy or nonmalignancy, resulted in a diagnosis as accurate as possible.

### Tools for improvement

The “Lynn & Adamson” model^27^ and the “Boyd-Murray” model^28^ are potential bases for improving end-of-life care. They propose a gradual transition from curative care to palliative care ending in end-of-life care and bereavement. Early continuous monitoring of a patient’s functioning is a prerequisite for giving the right care at the right moment. The kind of care needed by patients and relatives should be leading.^29^ Notwithstanding, Sercu and co-authors^30^ have shown how difficult it may be to apply theoretical concepts to clinical practice by exploring the advanced-terminal illness trajectories in 50 patients.

Various tools have been developed to assist physicians to time end-of-life care. Their value has increased as we found that the sole use of life expectancy is a poor basis for the timing of end-of-life care. The Palliative Performance Scale (PPS) and Palliative Prognostic Index (PPI) have proven usefulness.^31–33^ Monitoring the PPS should begin in an early stage of the illness, paying attention to changes in its magnitude as they are associated with survival time.^34^ It is indeed crucially important to recognize key transitions in the illness trajectory.^28,30^ The instrument is validated in a number of languages, including Dutch. Other tools such as the RADPAC score,^35^ the Surprise Question,^36^ and the Supportive and Palliative Care Indicators Tool^37^ may contribute to an early recognition of patients with a limited survival.

The “surprise question” is a simple tool to timely identify those patients who could be approached for end-of-life conservations. They are “sick enough that dying within the next year would not be a surprise.” However, one has to respect the wish of many patients, even when sensitively approached, to not discuss their future and end-of-life care. A positive answer on the surprise question may serve as a starting point for recording of PPS scores on a regular basis.

Unfortunately, payment of health care is not always *in concerto* with its organization and delivery. The Dutch Ministry of Health, Welfare and Sport adopted the ‘Lynn & Adamson’model,^27,38^ but the Dutch health care insurance system still strictly distinguishes curative care and end-of-life care.

### Future research

Future research should reveal reasons why the majority of our patients received only a brief period of end-of-life care similar to that in the United States.^14–16^ A possible reason is the rapid functional decline in cancer patients,^39^ making it difficult to estimate life expectancy. The study of Teno and co-authors^40^ provides more interesting leads to formulate research questions.

Rising costs are an important issue in health care policy. Our results show how the current policy fails in providing the right care at the right moment, while controlling costs. Further research should investigate the quality of care and potential savings when the needs for care are closely monitored at an early stage of a life-limiting disease.

Reducing excessively long periods of end-of-life care would inevitably require to increase the capacity along the continuum of end-of-life care. Further research should therefore aim at developing models to calculate optimal capacity for end-of-life care.

## Conclusion

Results of our retrospective study suggest that life expectancy is a weak basis for the timing of end-of-life care. Survival after the onset of end-of-life care was extremely skewed to the right. Half of the patients died within 15 days. A small minority of the patients, barely 11%, received “end-of-life” care for more than 90 days, giving rise to the majority of costs. Gender, age, and diagnosis were significantly associated with survival; however, the effect size was small and the clinical relevance of these associations seems limited. Consequently, further research should evaluate whether potential tools may improve end-of-life care, while using the available resources efficiently.

### Data sharing statements

The national office Statistics Netherlands, i.e., CBS, collects a wide range of data about the Dutch population, including health care data that are available for investigation on request. Their use is subject to strict rules, preventing disclosure of personal data. A personal identification number uniquely identifies each person in each of the CBS databases. Consulting these databases is only possible on the CBS computers using software provided by CBS. Analysis and results must be approved by CBS before publication, but authors remain fully responsible for accurate handling of data.

Data that support the findings of this study cannot be obtained from the authors as only CBS can make its nonpublic microdata accessible under strict conditions for statistical and scientific research. We therefore refer to https://www.cbs.nl/en-gb/our-services/customised-services-microdata/microdata-conducting-your-own-research. For further information, microdata@cbs.nl

## Notes

### Abbreviations Used

CBS: Statistics Netherlands
CI: Confidence Interval
EMEA: Exceptional Medical Expenses Act
HR: Hazard Rate
ICD: International Classification of Diseases
IQR: Interquartile Range
PH: Proportional Hazard
PPI: Palliative Prognostic Index
PPS: Palliative Performance Scale
RADPAC: Radboud indicators for Palliative Care needs
SD: Standard Deviation
SPSS: Statistical Package for Social Sciences
WMO: Medical Research Involving Human Subject Act
